# Zyxin Gene Expression in Patients with Varying Degrees of Coronary Artery Disease

**DOI:** 10.3390/ijms26157072

**Published:** 2025-07-23

**Authors:** Joanna Głogowska-Ligus, Józefa Dąbek, Agata Wypych-Ślusarska, Klaudia Oleksiuk, Karolina Krupa-Kotara, Ewelina Sobecko, Elżbieta Czech, Jerzy Słowiński

**Affiliations:** 1 Department of Epidemiology, Faculty of Public Health in Bytom, Medical University of Silesia in Katowice, 40-055 Katowice, Poland; awypych@sum.edu.pl (A.W.-Ś.); koleksiuk@sum.edu.pl (K.O.); kkrupa@sum.edu.pl (K.K.-K.); jslowinski@sum.edu.pl (J.S.); 2 Department of Cardiology, Faculty of Health Sciences in Katowice, Medical University of Silesia in Katowice, 40-055 Katowice, Poland; jdabek@sum.edu.pl; 3 Department of Biostatistics, Faculty of Public Health in Bytom, Medical University of Silesia in Katowice, 40-055 Katowice, Poland; ewelina.sobecko@sum.edu.pl (E.S.); emczech@sum.edu.pl (E.C.)

**Keywords:** zyxin, acute coronary syndrome, stable coronary artery disease

## Abstract

Acute coronary syndrome (ACS) remains the leading cause of mortality in developed countries. Although recent advances have improved our understanding of the pathophysiology of ACS and its primary consequence, myocardial infarction, many questions remain regarding the molecular and cellular changes occurring during and after an infarction. This study aimed to evaluate the expression levels of the zyxin (ZYX) gene in patients with ACS, stable coronary artery disease (stable CAD), and healthy controls. RNA was extracted from PBMCs and analyzed by quantitative real-time PCR (qRT-PCR). Gene expression was measured using TaqMan Gene Expression Assays and the number of ZYX mRNA molecules was quantified based on qRT-PCR kinetics. Kruskal–Wallis was used to compare gene expression levels among the three groups. A significantly higher number of ZYX gene copies was observed in both the ACS and stable CAD groups than in healthy controls (*p* < 0.0001 and *p* < 0.001, respectively). A statistically significant difference was also observed between the ACS and stable CAD groups (*p* = 0.004). The increased expression of zyxin observed in patients with ACS and stable CAD may reflect cellular repair mechanisms activated in response to myocardial injury.

## 1. Introduction

Mutual adhesion of cells as well as to the elements of extracellular matrix and effective communication between cells all points to the key role of adhesion processes in physiology (embryogenesis, wound healing) and pathophysiology (inflammation, oncogenesis and metastasis). These phenomena are linked to the migration of cells to target tissue sites or the ability of cells to separate from each other. In both cases, the underlying process involves disturbances or purposeful regulation of adhesion processes [1,2]. The ability of cells to organize their internal content, change shape, migrate and adhere, undergo signal transduction, and grow depends on a network of protein filaments located inside the cytoskeleton. Under physiological conditions, the cells of an organism are in a homeostatic equilibrium. This preserves the integrity of the closed high-pressure circulatory system following mechanical stress borne by the myocardium during infarction.

Damage to the vessel wall causes accumulation of adhesion molecules on the surface and alterations in the cytoskeleton, as well as changes in the interactions between cells and the extracellular matrix. The main adhesion-related receptors participating in contact between cells and the extracellular matrix are integrins [3]. Their expression occurs in all elements of the blood circulatory system: blood vessels, blood cells, myocytes, and smooth muscle cells. Integrin-dependent cell adhesion is linked with zyxin, a phosphoprotein localized on fibroblasts at cell adhesion foci. Zyxin contains a proline-rich N-terminal domain, and three LIM domains localized at the C-terminus. The latter is responsible for protein–protein interactions [4]. Studies in mouse models have shown that zyxin protects cardiomyocytes from apoptosis by interacting with actin [5,6].

Research has shown that zyxin is localized to focal adhesions and functions as a mechanosensory element in response to mechanical stimuli. It contains proline-rich regions that interact with the Ena/VASP family proteins. It may also influence the release of the von Willebrand factor by the endothelium, thereby modulating the organization of actin filaments around secretory granules [7]. Research indicates that zyxin regulates cell morphogenetic movements by influencing actin cytoskeleton dynamics and is essential for actin reorganization during cell migration [8]. Moreover, one study suggested an important role of zyxin in tissue formation, including heart valves and septa, in embryonic development [9].

The present study aimed to assess the transcriptional activity of the zyxin gene in patients with varying degrees of coronary artery disease and in healthy volunteers.

## 2. Results

The characteristics of the study group, considering sex, age, and laboratory results are presented in Table 1.

The whole study group (n = 150) included 75 subjects with acute coronary syndrome, 45 patients with stable coronary artery disease, and 30 healthy volunteers (control). The experimental group included 50 women (41.7%) and 70 men (58.3%) aged 30–86 (average 59.3 ± 11.1), whereas the control group consisted of 14 women (46.7%) and 16 men (53.3%) aged 43–73 (average 55.43 ± 7.50).

In the examined group, biochemical tests were performed including total cholesterol and its lipoprotein fractions, glucose, creatinine levels, and markers of myocardial necrosis: cardiac troponin T and creatine kinase—myocardial band (CK-MB) isoenzyme. General characteristics of the group studied are presented in Table 1.

In the studied group of patients with stable coronary artery disease and acute coronary syndrome, a more unfavorable lipid profile was found (higher concentration of total cholesterol, LDL fraction, triglycerides, and lower concentration of HDL cholesterol) compared to healthy volunteers (control group). The results obtained correlate with those obtained by other researchers [10,11]. All subjects had normal creatinine levels.

**Figure 1:**
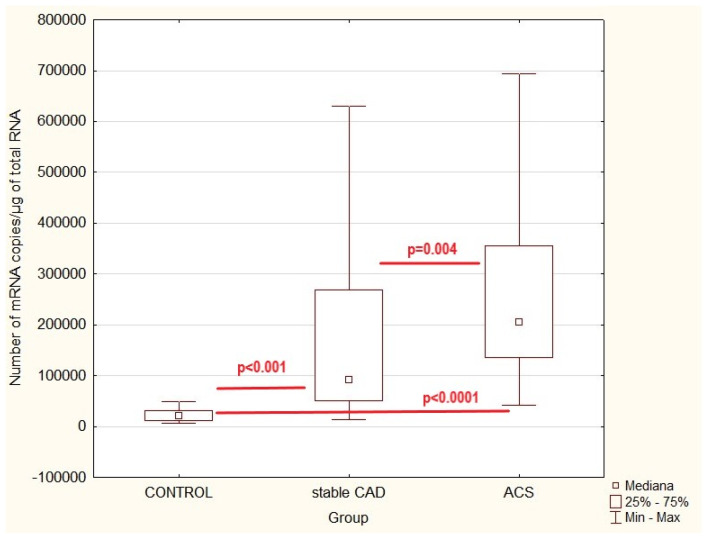
Zyxin gene expression values in patients with acute coronary syndrome (ACS) or stable coronary artery disease (stable CAD) and controls (healthy volunteers)—multiple comparison; *p*—statistical significance (multiple correlation).

To compare the expression profile of ZYX adhesion molecules in patients with acute coronary syndrome or stable coronary artery disease and controls, quantitative mRNA analysis was carried out (Figure 1). To compare the parameters investigated the Kruskal–Walli’s test (H) was used. *p* < 0.05 was considered statistically significant.

Statistical analysis demonstrated a significantly higher number of ZYX gene copies in acute coronary syndrome and in stable coronary artery disease as compared to controls (H = 70.99; *p* < 0.001). Multiple correlation analysis showed a high correlation between control and stable CAD (z = 5.07; *p* < 0.001) and control and ACS (z = 8.41; *p* < 0.0001). A statistically significant difference was also observed between the ACS and stable CAD groups (z = 3.21; *p* = 0.004).

**Figure 2:**
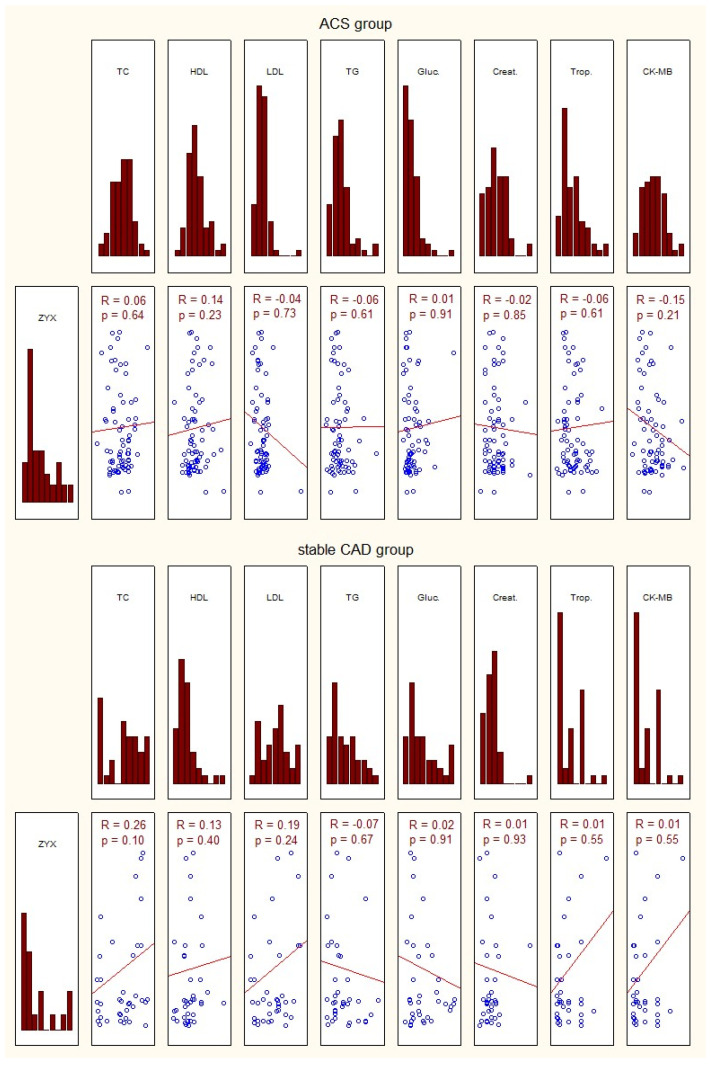

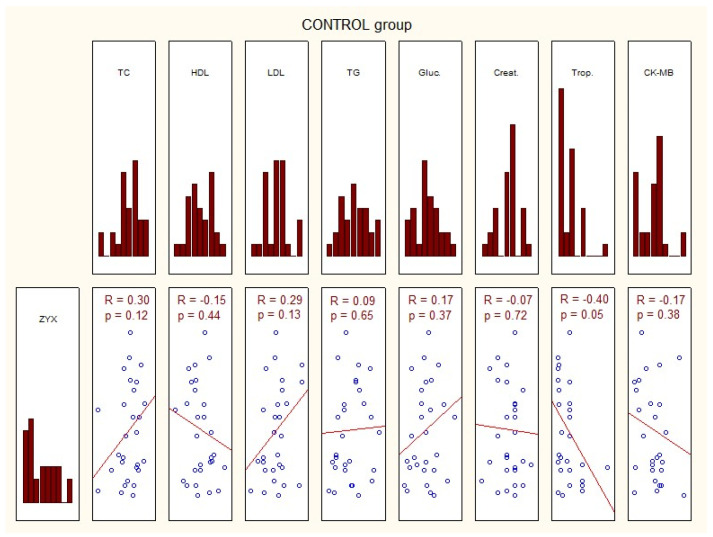
Characteristics of the investigated group in terms of expression of zyxin and basic laboratory tests. ACS—acute coronary syndrome; ZYX—zyxin; TC—total cholesterol; HDL—high-density lipoprotein; LDL—low-density lipoprotein; TG—triglycerides; Gluc.—glucose concentration; Creat.—creatinine; Trop.—troponin; CK-MB—creatine kinase—myocardial band; *p*—statistical significance; R—Spearman’s rank correlation.

The characteristics of the study group of patients with acute coronary syndrome, those with stable coronary artery disease, and controls, considering the transcriptional activity of zyxin, and biochemical tests are presented in Figure 2.

No correlation was found between zyxin gene expression in the study group and the basic laboratory tests (Figure 2).

## 3. Discussion

Acute coronary syndrome is the leading cause of death in developed countries [12]. Despite some progress in recent years in the understanding of the pathomechanism of acute coronary syndromes and myocardial infarction as their effect, there remain many unanswered questions concerning changes occurring during and after infarction. Research concerning factors that may influence the course of acute coronary syndrome and its aftermath can result in earlier diagnosis and treatment aimed at key features of the underlying pathomechanism [13]. Hemodynamic disturbances that occur in the damaged myocardium have been studied for decades. It is known that the underlying processes occur not only at the cellular level but also involve mediators. Complex intercellular interactions involving morphotic elements of the blood and inflammation mediators, interleukins, and integrins affect repair processes taking place in the infarcted myocardium [14].

In this study, we investigated the expression level of the integrin-dependent protein zyxin in patients with ACS and stable CAD during the first 24 h of hospitalization. Zyxin, as suggested by our previous inflammation-oriented studies, may be one of the proteins participating in the early response of myocardial cells just post-infarction [15]. As the latter causes damage to the myocardium and triggers the death of cardiomyocytes, the growing necrotic area starts a cascade of complex reactions involving the arterial lumen and molecules of the arterial wall. In this way, the integrity of the actin cytoskeleton is disturbed, and its cellular components begin to translocate. One of the proteins involved in this vessel remodeling process is zyxin. This protein moves from the cell surface to the nucleus and is responsible for cell spreading. In this way, alterations in zyxin-related gene expression are arranged. Cell spread and adaptation to new conditions appears to be an answer to endothelial dysfunction. Zyxin itself appears to be a potential therapeutic target at the stage of early changes in the endothelial cell phenotype [16].

The results of this study demonstrate a statistically higher number of mRNA copies of the zyxin gene in patients with ACS and stable CAD as compared to the results for healthy subjects. Moreover, our investigation showed statistically higher levels of zyxin gene expression in patients with ACS than in those with stable CAD. We also showed that an increase in zyxin gene expression was not correlated with laboratory test results, including parameters such as total cholesterol, glucose fractions, and creatinine concentration. Elevation of this gene expression in patients with myocardial infarction is most likely the result of a wound-healing process and the formation of a healed infarct. Our results correlate with those of Sperry et al. [17], who reported the role of zyxin in the controlled translocation of epithelial cells. They also suggested the zyxin-supported collective translocation of cells, which does not involve the loss of cell-to-cell junctions. Han et al. suggested the role of zyxin in cytoskeletal remodeling triggered by thrombin by circulating vasodilator-stimulated phosphoprotein. In addition, these investigators confirmed the key role of zyxin in the thrombin-dependent signaling pathway in endothelial cells. Broken interaction between the PAR-1 receptor and zyxin halts thrombin-induced tension of actin filaments. This hypothesis is corroborated by studies in which a similar phenomenon was observed when zyxin expression was blocked using siRNA [18].

Kato et al. found that cardiomyocyte survival is regulated by atrial natriuretic peptide (ANP) through cGMP-dependent nuclear accumulation of zyxin and actin. The accumulation of both molecules may represent a fundamental mechanism that facilitates nuclear signal transduction and increases cell survival [19,20]. Experimental animal studies have demonstrated the role of zyxin in the regulation of re-reendothelialization after carotid artery injury. Kang et al. demonstrated that zyxin is a new player in endothelial repair; thus, zyxin-mediated signaling may be a potential treatment target for vascular diseases.

Experimental animal studies have elucidated the role of zyxin in the regulation of re-reendothelialization following carotid artery injury. Kang et al. identified zyxin as a novel contributor to endothelial repair, suggesting that zyxin-mediated signaling may serve as a potential therapeutic target for vascular diseases [21]. This observation may hold significance in the context of coronary artery disease (CAD), as rapid endothelial restoration or re-reendothelialization is associated with reduced neointima and plaque formation following vascular injury [22]. Mori et al. hypothesized that an increase in zyxin expression may represent a mechanism for the repair and regeneration of damaged myocardium [9].

Adhesion foci-localized zyxin participates in several cellular interactions. It shares not only adhesion regulation processes but also signal transduction from the cytosol to the inside of the cell nucleus, which affects the activities of genes. This token participates in the regulation of pathways linked to proliferation and differentiation processes. Finally, zyxin accumulation in the cell nucleus plays an anti-apoptotic role, whereas, together with actin, it is involved in myocardial cell repair processes [23].

### 3.1. Strengths and Limitations of the Study

The strength of this study is its innovative nature, based on assessing the expression of the zyxin (ZYX) gene at various stages of coronary artery disease, which may contribute significantly to the understanding of the molecular mechanisms of heart muscle repair after injury. Additionally, the use of a sensitive quantitative PCR method and analysis of samples collected within the first 24 h of hospitalization allowed us to capture early changes in gene transcription. Careful patient selection and exclusion of confounding factors, such as active inflammatory states or neoplastic diseases, increase the reliability of the obtained results. An unquestionable advantage is the comparison of groups with acute coronary syndrome, stable coronary artery disease, and healthy volunteers, which enables a thorough analysis of differences in ZYX expression.

However, this study had some limitations. These include a relatively small sample size, especially in the control group, which may have affected the statistical power of the conclusions. Another limitation is the lack of long-term observations, which would allow for the assessment of changes in ZYX expression over time and their potential prognostic significance. Despite demonstrating significant differences between the groups, the absence of correlation between gene expression levels and the results of basic laboratory tests may indicate the need for more in-depth functional analysis and expansion of the panel of investigated markers. Another limitation is the lack of clear data regarding the medications taken, which could affect the expression level of the analyzed gene. Finally, owing to the observational nature of the study, the results do not allow for a definitive determination of a causal relationship between ZYX expression levels and the pathogenesis of coronary artery disease.

### 3.2. Practical Implications and Directions for Further Research

The practical implications stemming from this study suggest that increased zyxin expression may serve as a potential biomarker for early repair processes occurring in the cardiac muscle of patients with acute coronary syndrome and stable coronary artery disease. This could be significant in the context of predicting the course of the disease and assessing the effectiveness of therapy, especially during the tissue regeneration stage following heart attack. Identifying ZYX as an element activated in response to cardiac muscle injury also opens new therapeutic possibilities. Targeting the pathways regulated by this gene may contribute to the development of new treatment strategies aimed at improving endothelial regeneration and preventing the further progression of atherosclerosis.

Directions for further research should include the analysis of ZYX expression in a broader patient population, considering factors such as age, sex, the presence of comorbidities, and medications taken. It would also be worthwhile to conduct longitudinal studies to assess the dynamics of changes in the expression of this gene at different stages of the disease and its relationship with clinical prognosis. It is also important to expand research to include functional molecular analyses to better understand the role of zyxin in cellular signaling processes, vascular remodeling, and resistance to apoptosis. Integrating ZYX expression results with other biomarkers and medical imaging could contribute to the development of comprehensive diagnostic-prognostic models that support therapeutic decision-making in clinical practice.

## 4. Material and Methods

The study project was designed according to good clinical practice (GCP) rules consistent with the Declaration of Helsinki. The project was positively evaluated (decision BNW/NWN/0052/KB1/36/I/21/23) by the Bioethics Commission of the Medical University of Silesia in Katowice.

The study involved 150 subjects: 120 patients with varying degrees of coronary artery disease who were successively admitted to the Cardiology Clinic, Medical University of Silesia, in Katowice, and 30 healthy volunteers.

The inclusion criteria for this study were aged over 18 years, providing written, informed consent to participate in the study and consent to undergo coronary angiography, and the inclusion criterion for the group with stable coronary artery disease and the group with acute coronary syndrome was confirmation of the above diagnoses in imaging studies (coronary angiography, ultrasound), laboratory tests (morphology, blood smear, ESR, lipidogram, glycaemia, creatinine, bilirubin, electrolytes, necrosis markers), ECG (changes in the ST-T segment and T wave), and in the subjective examination (discomfort, chest pain, impaired exercise tolerance).

The exclusion criteria were a lack of patient consent, active inflammatory condition (e.g., infectious endocarditis, pneumonia, exacerbation of chronic obstructive pulmonary disease, etc.), chronic inflammatory diseases (e.g., collagenases), active neoplastic disease, advanced renal failure, and difficult contact with the patient.

The study material consisted of RNA isolated from peripheral blood mononuclear cells.

### 4.1. PBMC Isolation and RNA Extraction

Peripheral blood obtained from patient’s basilic vein in the first 24 h of hospitalization was used to obtain mononuclear cells separated with Ficoll Paque-Plus (GE17-1440-03 Merck, Darmstadt, Germany). Blood was gently layered on Ficoll at a 2:1 ratio and centrifuged for 40 min at 400× *g* at 40 °C. The lymphocyte layer was suspended in PBS and centrifuged again for 10 min at 400× *g* at 40 °C. This procedure was repeated twice. RNA was isolated using TRIzol (15596-018 Alab, San Jose, CA, USA) and modified Chomczyński and Sacchi method [24].

### 4.2. Reverse Transcription Quantitative Real-Time PCR

Transcriptional activity of the examined gene was assessed using a commercially available kit (TaqMan Gene Expression Assays 4331182 Hs 00170299_m1, ThermoFisher, Waltham, MA, USA). The number of ZYX mRNA molecules was determined based on QRT–PCR reaction kinetics using ABI PRISM™ 7000 sequence detection system (Applied Biosystems, Waltham, MA, USA) and a kit containing fluorescent dye (ROX QuantiTect Probe RT-PCR, Qiagen, Hilden, Germany).

QRT-PCR was performed in one step using a reaction mix containing 25 μL 2x QuantiTect Probe RT-PCR Master Mix (HotStarTaq DNA Polymerase, QuantiTect Probe RT-PCR buffer containing Tris-HCl, KCl, (NH_4_)_2_SO_4_, 8 mM MgCl_2_, pH = 8.7, dNTP mix, ROX reference dye), 0.5 μL QuantiTect RT Mix (Omniscript Reverse Transcriptase, Sensiscript Reverse Transcriptase in commercially available concentrations), and 1 μL of starter mix and TaqMan Gene Expression Assay probes (Applied Biosystems), RNA template, and pyrogen-free water. Together with the examined gene, commercially available DNA standards (β-actin, Applied Biosystems) were amplified. The amplification reaction mix for standards contained 25 μL 2x QuantiTect Probe RT-PCR Master Mix (HotStarTaq DNA Polymerase, QuantiTect Probe RT-PCR buffer containing Tris-HCl, KCl, (NH_4_)_2_SO_4_, 5 mM MgCl_2_, pH = 8.7, dNTP mix, ROX reference) (Qiagen GmbH) and 0.5 μM sense and antisense starters, β-actin cDNA template, and pyrogen-free water. The reverse transcription reaction was carried out at 50 °C for 30 min. After initial activation of HotStar Taq DNA Polymerase at 95 °C for 15 min, a two-stage reaction was conducted: 94 °C for 15 s (denaturation), 60 °C for 60 s (starter annealing). Final elongation of the amplification products was carried out at 72 °C for 10 min.

### 4.3. Statistical Analysis

The expression of the studied gene was inferred based on the number of mRNA copies per 1 µg of total RNA. Statistical analyses were performed using Statistica v.13.0. To assess changes in the examined parameters, in the first stage, the distribution of variables was checked using the Shapiro–Wilk test. Statistical analysis was performed using the Kruskal–Wallis test. The level of statistical significance was set at *p* ≤ 0.05. The correlation between the values of ZYX gene expression and total cholesterol and its lipoprotein fractions and glucose and creatinine levels for the investigated group was verified by calculating Spearman’s R correlation coefficient.

## 5. Conclusions and Suggestions

The results of our study showed a significantly higher expression of zyxin (ZYX) in patients with acute coronary syndrome (ACS) and stable coronary artery disease (CAD) than in healthy individuals. Additionally, ZYX expression levels were higher in patients with ACS than in those with stable CAD, which may indicate its involvement in early cellular response mechanisms to myocardial injury. The lack of correlation between the expression levels and biochemical parameters suggests that ZYX may act independently of traditional cardiac markers. The increased transcriptional activity of ZYX may reflect reparative and remodeling processes of the endothelium after an ischemic event; however, further research is necessary to confirm its value as a potential biomarker or therapeutic target.

## Figures and Tables

**Table 1:** Characteristics of the studied group include laboratory results.

Variable		Studied Group				
		C/stable CAD/ACS n = 150	C n = 30	Stable CAD n = 45	ACS n = 75	Test p
Sex	Woman	n = 64; 42.7%	n = 14; 46.7%	n = 20; 44.4%	n = 30; 40.0%	χ^2^ = 0.47 p = 0.79
	Man	n = 86; 57.3%	n = 16; 53.3%	n = 25; 55.6%	n = 45; 60.0%	
Variable		Me	Me	Me	Me	
		IQR	IQR	IQR	IQR	
Age [years]		58.00	52.50	62.00	58.00	H = 4.96
		14.00	12.00	15.00	15.00	NS
Total cholesterol [mg/dL] (N: 115–190)		185.00	170.50	175.00	199.00	H = 24.89
		44.00	21.00	61.00	54.00	p < 0.001
HDL cholesterol [mg/dL] (Women N: >45) (Men N: >40)		44.00	56.50	37.00	46.00	H = 33.86
		19.00	17.00	7.00	17.00	p < 0.001
LDL cholesterol [mg/dL] (N: <115)		118.50	108.50	113.00	125.00	H = 7.28
		41.00	46.00	67.00	47.00	p = 0.03
Triglycerides [mg/dL] (N: <150)		116.00	96.50	114.00	123.00	H = 7.53
		57.00	39.00	50.00	70.00	p = 0.02
Glucose [mg/dL] (N: 74–106)		112.50	89.00	109.00	128.00	H = 52.06
		48.00	12.00	45.00	56.00	p < 0.001
Creatinine [mg/dL] (N: 0.67–1.17)		1.00	1.00	1.05	1.00	H = 12.60
		0.20	0.10	0.20	0.30	NS
Troponin T [ng/mL] (Women N: <0.014) (Men N: <0.022)		0.12	0.01	0.01	2.12	H = 112.21
		2.11	0.01	0.02	2.07	p < 0.001
CK-MB [ng/mL] (Women N: <3.61) (Men N: <4.87)		4.32	2.90	0.01	7.49	H = 125.28
		7.47	0.80	0.02	2.35	p < 0.001

C—control; stable CAD—stable coronary artery disease; ACS—acute coronary syndrome; CK-MB—creatine kinase—myocardial band; n—sample size; Me—median; IQR—interquartile range; N—standard in laboratory tests; χ^2^—Chi square test result; H—Kruskal–Wallis test result; *p*—statistical significance; NS—not significant.

## Data Availability

All data can be obtained from the corresponding author upon reasonable request.
